# Does Poultry Consumption Increase the Risk of Mortality for Gastrointestinal Cancers? A Preliminary Competing Risk Analysis

**DOI:** 10.3390/nu17081370

**Published:** 2025-04-17

**Authors:** Caterina Bonfiglio, Rossella Tatoli, Rossella Donghia, Pasqua Letizia Pesole, Gianluigi Giannelli

**Affiliations:** 1 Unit of Data Science, National Institute of Gastroenterology—IRCCS “Saverio de Bellis”, Castellana Grotte, 70013 Bari, Italy; rossella.donghia@irccsdebellis.it; 2 Core Facility Biobank, National Institute of Gastroenterology IRCCS “Saverio de Bellis”, Castellana Grotte, 70013 Bari, Italy; letizia.pesole@irccsdebellis.it; 3 Scientific Direction, National Institute of Gastroenterology—IRCCS “Saverio de Bellis”, Castellana Grotte, 70013 Bari, Italy; gianluigi.giannelli@irccsdebellis.it

**Keywords:** poultry, meat, mortality, competing risk

## Abstract

**Background**: Poultry meat is currently among the most widely consumed meats in Italy and worldwide. Poultry is reasonably affordable and accessible, explaining the high global consumption rates. This population-based prospective cohort study investigated the association between meat consumption and gastrointestinal cancers (GCs) and other causes of mortality in southern Italy. **Methods**: Data were collected from 4869 participants in the MICOL and NUTRIHEP cohorts. The EPIC questionnaire was used to elicit information on food and drink consumption. For analytical purposes, weekly meat consumption was grouped into four categories: total meat: <200 g, 201–300 g, 301–400 g, and >400 g red meat: <150 g, 150–250 g, 251–350 g, and >350 g; poultry: <100 g, 100–200 g, 201–300 g, and >300 g. Cox proportional hazard regression and competing risk models were employed for statistical analysis. **Results**: Analyzing weekly poultry consumption, it was observed that subjects consuming more than 300 g had a 27% higher risk of death from all causes [HR 1.27; 95% CI (1.00; 1.61)] than those consuming less than 100 g. In addition, for GCs, the SHR for weekly poultry consumption above 300 g was 2.27 [95% CI (1.23; 4.17)], a risk that for men increased to 2.61 [95% CI (1.31; 5.19)]. **Conclusions**: Our study showed that poultry consumption above 300 g/week is associated with a statistically significant increased mortality risk both from all causes and from GCs. The risk is higher for men than for women.

## 1. Introduction

Although red and processed meat consumption continues to increase in the United States and Western countries, there has recently been a shift toward a higher percentage of white meat intake [1], not only in the United States but also in Italy [1]. The ISMEA report from 2013 showed a world poultry consumption per capita of 13.3 kg and an expected increase of around 19% by 2022. In Italy, per capita poultry consumption has increased by 8.5% over the last decade from 11.7 kg to the current 12.7 kg. Poultry is reasonably affordable and accessible, explaining the high global consumption rates [2].

The increased appreciation of the population for these types of meat has strongly stimulated the activity of the processing industry.

The Dietary Guidelines for Americans, 2020–2025 (DGA), define poultry as all forms of chicken, turkey, duck, geese, guineas, and game birds (e.g., quail and pheasant) [3]. The healthy eating guidelines indicate 100 g as a standard portion of poultry, suggesting its consumption one to three times a week [4]. The DGA defines poultry as a noble food because it provides protein of high biological value and is often lower in fat than meat products from other animal sources [2]. For example, chicken breast is 93% protein and contains only 7% fat [4]. The consumption of lean and fresh cuts is recommended, while processed products should be limited [4]. Industrial processing may involve the addition of sodium, saturated fat, sugar, and preservatives, making poultry less healthy [5,6].

The consumption of poultry is often considered a healthier alternative to red meat, even though the population’s eating habits often deviate from the guidelines [2,7]. In fact, 26% of the chicken meat consumed is in the form of prepared and processed products (hamburgers, kebabs, rolls, bites), 61% of the product as parts, and only 13% as whole chicken [1].

In 2015, the International Agency for Research on Cancer defined meat as “carcinogenic to humans” and red meat as “probably carcinogenic to humans” [8,9]. In the 2018, the World Cancer Research Fund and the American Institute for Cancer Research stated that there is strong evidence regarding the association between the consumption of meat, red and processed, and cancer risk [10].

Furthermore, only limited studies in the literature support poultry as being inversely associated with the risk of all causes of mortality [11,12,13].

The aim of this study was to evaluate for the first time the association between white meat consumption and mortality from all causes and from gastrointestinal cancers, focusing on the effects of poultry consumption.

We hypothesized that there would be a significant association between white meat consumption and the risk of death both from all causes and from gastrointestinal cancer.

## 2. Materials and Methods

### 2.1. Study Population

The details about the study population have been documented in previous publications [14,15]. The MICOL study [16,17] is a population-based prospective cohort study that was randomly selected from the electoral rolls of Castellana Grotte (participants aged 30 and above) in 1985 and has undergone three follow-up phases: 1992–1993, 2005–2006, and 2017–2019. This analysis pertains to the second follow-up (2005–2006), during which a random sample of individuals aged 30–50 (PANEL study) was incorporated into the initial cohort to compensate for aging.

The NUTRIHEP study is a cohort formed in 2005–2006 using the registries of general practitioners in Putignano (participants aged 18 and older). The sex and age distributions in these registries are assumed to mirror those of the general population.

**Figure 1:**
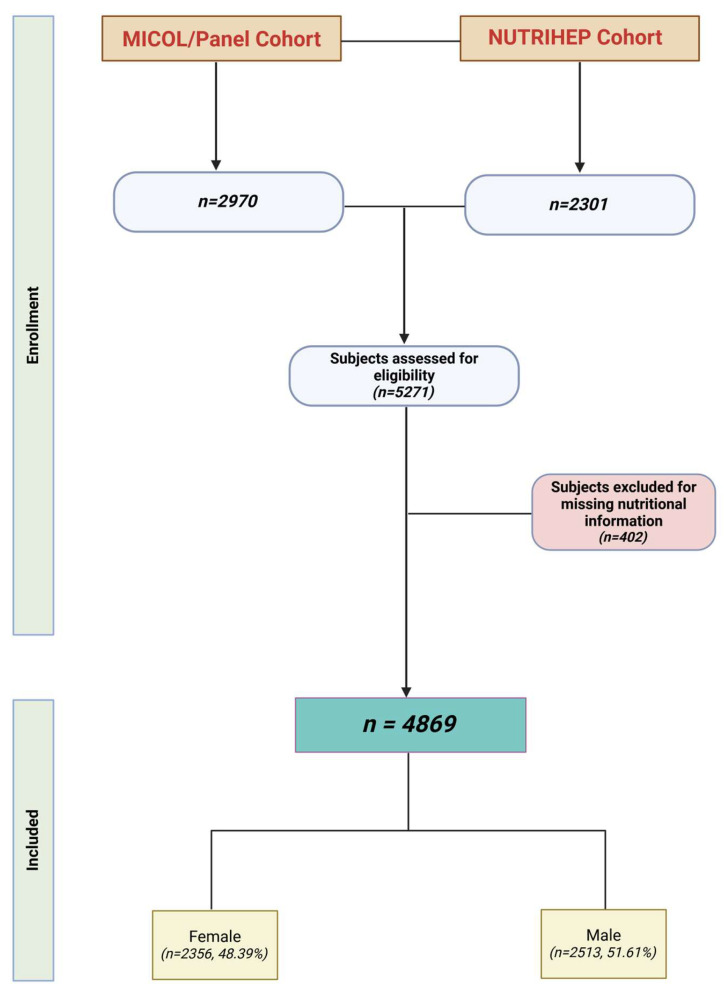
Flowchart of study population (created using Biorender.com).

Of 5378 eligible subjects, 5271 (98.1% response rate) provided written informed consent to participate in this study. Those who did not complete the lifestyle and diet questionnaires were excluded: 111 from MICOL and 291 from NUTRIHEP (see Figure 1).

All procedures adhered to the ethical guidelines established by the institutional research committee (National Institute of Gastroenterology, IRCCS “S. De Bellis” Research Hospital), following the ethical approval for the MICOL study (DDG-CE-589/2004, 18 November 2004) and the NUTRIHEP study in 2005 (DDG-CE-502/2005, 20 May 2005). This study was conducted in alignment with the 1964 Declaration of Helsinki and its later revisions, prior written informed consent being obtained from each participant.

### 2.2. Data Collection

Participants were interviewed by medical personnel to gather details about their sociodemographic characteristics, health status, personal history, and lifestyle factors, including tobacco use (ever or currently), eating habits, and educational level (illiteracy, elementary school, secondary school, high school, and university degree) [18]. Employment status was classified into the following categories: pensioners and unemployed, managers and professionals, craft, agricultural, and sales workers, homemakers, and elementary occupations [19]. Marital status was categorized as single, married/coupled, separated/divorced, or widowed/er. Weight was measured with subjects wearing only underwear and standing on a SECA^®^ electronic scale, with results rounded to the nearest 0.1 kg. Height was recorded using a wall-mounted SECA^®^ scale and rounded to the nearest cm. Blood pressure (BP) was measured according to international protocols [20,21], using the average of three readings. A validated dietary questionnaire was administered to assess the usual food intake, with assistance from trained nutritionists [22]. This included the European Prospective Investigation on Cancer (EPIC) Food Frequency Questionnaire (FFQ), and individual nutrient contributions were calculated from the foods listed in the dietary questionnaires using the standardized EPIC Nutrient Database [23,24].

The European Prospective Investigation on Cancer (EPIC) Food Frequency Questionnaire (FFQ) [22] was administered by trained nutritionists to estimate the usual food intakes. Individual nutrient intakes were derived from foods included in the dietary questionnaires through the standardized EPIC Nutrient Database [23,24]. The EPIC FFQ input was performed online, and centralized processing was carried out by the National Cancer Institute, based in Milan.

### 2.3. Outcome Assessment

Participants were followed until 31 December 2024, and their vital status or emigration was verified through the registry offices of the Castellana Grotte and Putignano municipalities. Their mortality status was verified through the regional database (EDOTTO).

Information on causes of death from 2006 to December 2024 was extracted from the Apulian Regional Registry using the death certificate according to the WHO guidelines [25].

The causes of death were grouped based on ICD-10 code classifications: gastrointestinal cancer (GC) (codes C15-C26), other cancer (OCr) (ICD-10 C01-C014 and C30-C97), and other causes of death (DOC).

### 2.4. Exposure Assessment

Three groups of meat consumption exposure were used: total meat, red meat, and poultry.

The total meat group comprised lamb, pig, calf, and horse for red meat, and rabbit and poultry for white meat.

Consumption for each type of meat was divided into four categories based on weekly intake: <150, 150–250, 251–350, and >350 g for red meat; <100, 100–200, 201–300, and >300 g for poultry; and <200, 201–300, 301–400, and >400 g for total meat.

The categories were based on the optimal weekly consumption of red meat and poultry (the second category), and the amount was increased by 100 g per category to determine whether a trend existed [26].

### 2.5. Statistical Analysis

Data are presented as mean ± standard deviation (SD), mean and standard error (SE), or median (IQR) for continuous data, and frequency (%) for categorical data.

The observation time was from enrollment to death, moving elsewhere, or the end of the study (31 December 2024), whichever occurred first.

Since age is the most critical risk factor for death, we chose age at death as the time scale.

We set 94 years of age as the maximum observation age.

We used Cox proportional hazards regression with age as the underlying time metric to estimate hazard ratios (HRs) and 95% confidence intervals (CI) for the association between total meat, red meat, and poultry consumption and all-cause mortality.

Schoenfeld residuals were performed to test the proportional hazard assumption [27] (Figures S1–S3).

Initially, confounding variables were selected from the existing literature.

Then, the absolute minimum procedure (LASSO Cox) was used to reduce the number of candidate predictors and select those most helpful for building the final model [28] (see Figure S4).

We constructed six Cox models. The 1st model (a) was adjusted for sex (women vs. men), gamma-glutamyl transpeptidase (GGT), glucose, total cholesterol, smoking (bever vs. current), hypertension (absence of pathology vs. presence), diabetes (disease absence vs. presence), dyslipidemia (disease absence vs. presence), wine (mL/week), and the relative Mediterranean Scoring System (rMED). The 2nd model (b) was adjusted for sex (women vs. men), GGT, glucose, total cholesterol, smoking (never vs. current), hypertension (disease absence vs. presence), diabetes (disease absence vs. presence), dyslipidemia (disease absence vs. presence), wine (mL/week), rMED, and white meat consumption (g/week). The 3rd model (c) was adjusted for sex (women vs. men), GGT, glucose, total cholesterol, smoking (never vs. current), hypertension (disease absence vs. presence), diabetes (disease absence vs. presence), dyslipidemia (disease absence vs. presence), wine (mL/week), rMED, and red meat consumption (g/week). The 4th (d), 5th (e), and 6th (f) models were stratified by sex and adjusted as models (a), (b), and (c), respectively.

We used the lowest category as the reference group.

Flexible parametric survival models were run for subdistributions for cause-specific mortality using a competing risk approach [29,30].

This model satisfied the proportional hazard assumption for the subpopulation hazard being modeled, which means the general hazard ratio formula was essentially the same as for the Cox model, except for a minor difference in that the betas in the Cox model were replaced by gammas in Fine and Gray’s model. Consequently, we similarly interpreted the gammas for the betas estimated from the Cox model, except that it estimated the effect of specific covariates in the presence of competing events. The Fine and Gray model can also be extended for time-dependent covariates [31].

We estimated the subdistribution hazard ratio (SHR) for the three exposure categories associated with the risk of developing three competing events: gastrointestinal cancer, other cancer, and death from other causes.

We constructed six flexible parametric survival models in the same way as the Cox models.

We fitted the cause-specific cumulative incidence function (CIF) using post-estimation tools.

All statistical analyses were performed using Stata, Statistical Software version 18.0 (StataCorp. 2023. Stata Statistical Software: Release 18. College Station, TX, USA: StataCorp LLC).

## 3. Results

### 3.1. Participant Characteristics

During the observation period of 27,878.918 person-years, a total of 1028 participants (21.1%) died, resulting in an incidence rate of 36.87 per 1000 person-years. Among these deaths, 108 (10.5%) were due to GC: 28 to liver cancer, 22 to pancreatic cancer, and 37 to colorectal cancer. An additional 180 deaths (17.5%) were attributed to other cancers, including 45 due to lung cancer, 19 due to breast cancer, and 18 due to prostate cancer. The remaining 740 deaths (72.0%) were due to other causes: 31 to myocardial infarction, 37 to stroke, 18 to Alzheimer’s disease, and 62 to dementia.

Table 1 presents the main characteristics of the 4869 participants, categorized by sex.

The mean age of the 3841 participants still alive was 65.40 (±13.24) years, and the oldest subject was 105 years as of 31 December 2024.

The mean age at death was 81.09 years (±10.8), 82.71 (±10.27) for women and 79.94 (±10.84) for men.

Among them, 860 (17.7%) consumed less than 200 g of total meat per week, 906 (18.6%) consumed between 200 and 300 g per week, 870 (17.9%) consumed between 301 and 400 g per week, and 2717 (55.8%) consumed more than 400 g per week. The median rMED score was equal to eight across all participants (nine for women and eight for men), indicating good adherence to the Mediterranean diet.

### 3.2. Participant Meat Consumption

The 108 subjects who died from gastrointestinal cancers (GCs) had the highest weekly total meat consumption compared to the other causes of death: 411.74 (23.92) versus 407.90 (22.19) for different cancers (OCr) and 356.56 (9.79) for the remaining causes of death (DOC) (Table 2).

Higher consumption of red meat was observed among those who died from other causes of cancer (OCr) at 263.34 (18.49) compared to 230.22 (15.86) for gastrointestinal cancer and 208.25 (6.48) for the remaining causes of death.

The highest consumption of white meat, particularly poultry, was observed in deaths from GC at 136.65 (12.72) g/week, followed by deaths from OCr at 109.90 (8.10), and deaths from DOC at 104.69 (4.55) (Table 2).

Overall, for the 1028 deceased subjects, the percentage of red meat consumed out of the total weekly meat consumption was 59.3%, while for white meat, it was 40.7% (29.4% poultry only).

Regarding specific causes of death, among those who died from GC, the percentage of red meat consumption, as a proportion of total meat consumed during the week, was 56.0%, while for white meat, it was 44.0% (with poultry accounting for 33.3%).

The analysis of weekly red meat consumption, as a proportion of total meat, for those who died from OCr was 64.5%, whereas for white meat, it was 35.5% (27.0% when considering poultry only).

For other causes of death (DOC), the percentage of red meat consumption, compared to the total amount of meat consumed weekly, was 58.4%, while white meat accounted for 41.6% (with 29.5% poultry only).

Good adherence to the Mediterranean diet was observed for total mortality and each specific death category (see Table 2).

Table S1 shows the distribution of weekly meat consumption by type, stratified by sex and cause of death. It is observed that the highest poultry consumption was by men who died from GC causes, at 147.1 g/week (15.84), compared to 112.1 g/week (17.95) in women. Poultry consumption was similar between the sexes for both OCr [112.8 g/week (10.77) for men and 110.0 g/week (13.87) for women] and DOC [103.9 g/week (4.73) for men and 104.4 g/week (7.62) for women].

### 3.3. Meat Consumption and Cancer Mortality

Table 3 and Table 4 show the results of the mortality hazard ratios (HRs) and subdistribution hazard ratios (SHRs) according to weekly consumption of the various types of meat.

Table 3 shows a protective effect against all causes of death observed for individuals who consumed 200–300 g of meat per week [HR 0.81; 95% CI (0.67; 0.98)]. The same protective effect was observed for men who consumed the same amount of meat [HR 0.73; 95% CI (0.56; 0.95)] (Table 4).

Regarding red meat, a protective effect against all causes of death was observed for those who consumed 150–250 g per week [HR 0.80; 95% CI (0.65; 0.98)] (Table 3).

When analyzing weekly poultry consumption, subjects who consumed more than 300 g had a 27% higher risk of death from all causes [HR 1.27; 95% CI (1.00; 1.61)] compared to those who consumed less than 100 g (Table 3).

In other meat consumption categories, no statistically significant differences in overall mortality were found (Table 3 and Table 4).

Weekly consumption of 200–300 g of total meat for gastrointestinal cancers was associated with a 54% reduction in the risk of death [SHR 0.46; 95% CI (0.23; 0.93)] compared with those who consumed less than 200 g per week (Table 3). Among men, for the same consumption category, this reduction was 68% [SHR 0.32; 95% CI (0.13; 0.80)] (Table 4).

For gastrointestinal cancers, the SHR for weekly poultry consumption between 100 and 200 g/week was 1.65 [95% CI (1.06; 2.59)]; between 201 and 300 g/week, it was 2.11 [95% CI (1.09; 4.09)]; while above 300 g, it was 2.27 [95% CI (1.23; 4.17)] (Table 3). Among men, for weekly poultry consumption above 300 g, the SHR was 2.61 [95% CI (1.31; 5.19)] (Table 4).

No statistical significance was found for OCr and DOC in the models presented in Table 3 and Table 4.

**Figure 2:**
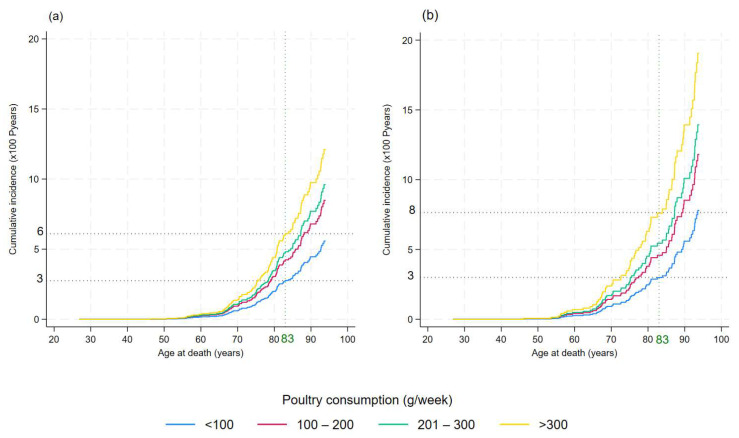
Cumulative incidence of gastrointestinal cancer mortality by poultry consumption category: <100 g/week, 100–200 g/week, 201–300 g/week, and >300 g/week. (**a**) Cumulative incidence functions of gastrointestinal cancer mortality plotted for four poultry consumption categories. (**b**) Cumulative incidence functions for gastrointestinal cancer mortality in men. This graph highlights how different levels of poultry intake affect the probability of death from gastrointestinal cancer, with separate lines for each consumption category.

The cumulative incidence of gastrointestinal cancer mortality by poultry consumption categories is shown in Figure 2.

The impact of poultry consumption on gastrointestinal cancer mortality became evident as early as age 65 and for men at age 60 (panels (a) and (b) in Figure 2, respectively).

At age 83 years, the mean age of death for the Apulian population, the probability of death from gastrointestinal cancers was 6% for those who consumed more than 300 g per week of poultry, compared with 3% for those who consumed less than 100 g per week (Figure 2a). In men, the probability of death at age 83 from gastrointestinal cancer was 8% for those who consumed more than 300 g per week of poultry, compared with 3% for those who consumed less than 100 g per week (Figure 2b).

## 4. Discussion

This study, conducted on a population of 4869 middle-aged Italian participants from Castellana Grotte and Putignano (Apulia, Italy), describes meat consumption and its impact on the risk of death from all causes and from cancers, especially gastrointestinal cancers.

To directly evaluate the effects of poultry consumption, we separated white meat from red meat intake. Our results show that consumption of more than 100 g/week of poultry was associated with an increased risk of death both from all causes and from GC. The risk increased progressively as the portion consumed increases and was greater when compared to the same portion of red meat.

There is little as well as conflicting information in the literature on this association we studied.

Some studies reported a possible positive effect of higher white meat consumption on gastric cancer risk but emphasized the need for further studies to confirm this association [32].

Other studies have reported no association between the intake of red meat, processed meat, fish, or poultry and the risk for colon or rectal cancer [33].

No association between poultry intake and colorectal cancer (CRC) risk was reported by the World Cancer Research Fund Continuous Update Project [34].

The Agency for Research on Cancer defined red meat as “*probably cancerogenic to humans*” [8], and the World Cancer Research Fund and the American Institute for Cancer Research spoke about strong evidence of red meat consumption and the risk of CRC [23], but poultry is not mentioned as a risk factor. Indeed, Shi et al. reported a reduction in the risk of CRC associated with increasing poultry consumption by 50 g/day in a meta-analysis that included prospective cohort studies from the United States, Australia, Europe, and Japan [35]. The protective effect observed with poultry consumption could be attributed to reduced red meat consumption in the diet. Compared to red meat, poultry is lower in saturated fat and heme iron [36], which is associated with oxidative stress due to its ability to generate free radicals and cause DNA damage [37,38].

Meat cooking and storage methods significantly influence the risk of developing gastrointestinal cancers. Boldo et al. reported results in line with our study, focusing on the cooking method [39]. They identified an increased risk of gastric and intestinal tumors among consumers who preferred well-done white or red meat. Furthermore, stewing and oven-baking appeared to be the cooking methods with the most significant effects on GAC for white meat [39].

Protein-rich foods, when cooked at high temperatures or heated for prolonged times, generate mutagenic compounds, including heterocyclic amines (HCAs), polycyclic aromatic hydrocarbons (PAHs) and N-nitroso compounds (NOCs) [40].

Chicken, in the breast cut, has a protein content of 23.3 g/100 g [41]. We can assume that white meat cooked at high temperatures or for a long period (e.g., griddling, barbequing, stewing) also forms high levels of mutagens, which could have an important role in GC pathogenesis [42].

Therefore, when studying the association between meat and cancer risk, an evaluation of meat type and cooking method should not be neglected [39].

An inverse association between poultry intake and total mortality was observed among low-meat consumers in Asia [43] and among American men [44]. Thus, according to these results, a high intake of red and processed meat but not poultry seems to be associated with CRC and mortality. However, further studies are needed to evaluate the relationship between meat consumption and diet quality. In our sample, despite good adherence to the Mediterranean diet model, people who died of GC consumed a higher amount of meat, particularly poultry.

In line with our results, Heddie et al. found a significant increase in CRC risk, but not all-cause mortality, for high versus low poultry intake. None of these associations were modified by increased adherence to healthy eating guidelines [45].

In our previous study, we showed how combining the consumption of red meat with a portion of broadleaf vegetables reduced the risks associated with meat consumption. This effect was not observed for white meat; despite our sample’s adherence to the Mediterranean diet, which includes the regular consumption of vegetables at every main meal, the risks remain unchanged. This leads us to suggest a different pathogenic mechanism for white meat than red meat, likely linked to the type of animal feed and how the animals are raised. However, further studies are required to support this hypothesis [46].

The consumption of poultry influences the risk of mortality differently in the two sexes. Our results showed that men have a higher risk than women of dying from GC for the same proportion of poultry consumed. Also, Yumie et al. identified a statistically significant sex difference in meat consumption and mortality [47].

Unlike in our case, this applied to red meat but not to poultry: in men but not in women, the consumption of red meat was associated with an increased risk of mortality, while consumption of poultry reduced it [47].

The factors that might explain the observed sex differences need clarification. To the best of our knowledge, in the scientific literature, there is no known biological mechanism to explain the observed sex differences [47].

We can assume that sex hormones have an effect. Studies conducted in mouse models have found estrogen-binding receptors throughout the gastrointestinal tract [48]. This leads us to hypothesize a role of estrogen not only in the different ability of the two sexes to metabolize nutrients but also in the risk of developing some diseases of different types between men and women [15]. Further investigation is needed to support this hypothesis.

The genetic component is important because some genes (e.g., TP53, APC) have now been widely demonstrated to be associated with tumor risk and modifiable by epigenetic mechanisms in both sexes, such as DNA methylation [49]. Furthermore, there are dietary differences between men and women. Men tend to consume more red meat, alcohol, fruit, and industrial food in greater quantities,; therefore, they are the subjects who adhere less to the Mediterranean diet. On the contrary, women prefer healthier foods, such as fruit and vegetables, whole foods, and yogurt and prefer smaller portions or those that are commensurate with their physiological needs [50].

The effect of poultry consumption on the risk of death from GC also differed according to age. In our sample, the risk of mortality from GC at the age of 60 was similar for those consuming less than 100 g/week and those consuming more than 300 g/week. At the age of 83, the average age of death in Apulia [51], the risk of death was twice as high in the highest consumption category as in the lowest. This difference was more pronounced for men. In men, the difference in mortality risk between various categories of poultry consumption was already evident before the age of 60. In addition, at the age of 83, a higher risk of death was already observed for men than for the total sample for those consuming less than 100 g/week.

This result further emphasizes the difference between the two sexes regarding the association between poultry consumption and mortality, identifying men as a category at greater risk. However, further studies are needed to investigate this association in more detail and confirm the finding.

### Strengths and Limitations

This study has several strengths but also some limitations. One of the strengths of our study is the sample size. It included 4869 participants from a cohort based on two municipalities in southeastern Italy. We also boasted an average follow-up of 19 years. Overall, this study is one of the few to have analyzed the effects of poultry on health status, particularly in relation to mortality risk.

There are also some limitations. One of these is the absence of information on the consumption of processed poultry and the form of processing (i.e., cold cuts or fast food). This is because the questionnaire used to assess eating habits only included a general question regarding poultry consumption. However, to the best of our knowledge, only 1% of articles identified in the scientific literature assessed the relations between poultry, processed poultry consumption, and human-health-related parameters [52,53]. Although the EPIC food questionnaire does not request the origin of meat farming, we can assert that, despite both cohorts being located in rural areas, the meat consumed—particularly poultry—was predominantly battery-farmed rather than locally sourced.

Another potential limitation is related to self-reporting of diet; however, to overcome this problem, each FFQ was reviewed by our expert nutritionists at the time of questionnaire delivery.

Our study did not include a measure of physical activity, a potentially serious limitation given previous research findings linking physical activity with all causes and cause-specific mortality. The only way to indirectly measure the physical activity could have been the International Physical Activity Questionnaire (IPAQ). However, this instrument has only been validated for the 18–69 age group, i.e., middle age [54], which is far from our mean age. Therefore, the absence of this information did not allow us to include this variable as a potential confounder in the model. Thus, we may have overestimated or underestimated the effect of diet due to a confounding or effect modification of physical activity [55]. The residual confounding was consistent across all models analyzed, and the differing environmental impacts could partly explain the inconsistencies with other studies [56].

## 5. Conclusions

Our study showed that white meat consumption above 300 g/week was associated with a statistically significant increased mortality risk from all causes and GC. The risk was higher for men than for women.

However, further studies are needed to confirm our findings and learn more about the effects of processed poultry.

In our opinion, it is important to learn more about the long-term effects of this food category, white meat, that is widely consumed by the world population who, perhaps mistakenly, consider it healthy in absolute terms.

We believe it is beneficial to moderate poultry consumption, alternating it with other equally valuable protein sources, such as fish. We also believe it is essential to focus more on cooking methods, avoiding high temperatures and prolonged cooking times.

## Figures and Tables

**Table 1:** Characteristics of participants by sex from MICOL/PANEL and NUTRIHEP studies. Castellana Grotte. Putignano (BA). Italy. 2005–2024.

Parameter ^1^	Total Sample ^2^	Sex ^3^		*p*-Value ^4^
		Women	Men	
N (%)	4869	2356 (48.39)	2513 (51.61)	
Total meat categories (g/week)				
<200	860 (17.7)	503 (58.5)	357 (41.5)	<0.001
200–300	906 (18.6)	466 (51.4)	440 (48.6)	
301–400	870 (17.9)	416 (47.8)	454 (52.2)	
>400	2717 (55.8)	1192 (43.9)	1525 (56.1)	
Red meat categories (g/week)				
<150	466 (9.6)	275 (59.0)	191 (41.0)	<0.001
150–250	1036 (21.3)	582 (56.2)	454 (43.8)	
251–350	1101 (22.6)	500 (45.4)	601 (54.6)	
>350	2266 (46.5)	999 (44.1)	1267 (55.9)	
Poultry categories (g/week)				
<100	2353 (48.3)	1188 (50.5)	1165 (49.5)	<0.001
100–200	1508 (31.0)	736 (48.8)	772 (51.2)	
201–300	520 (10.7)	233 (44.8)	287 (55.2)	
>300	488 (10.0)	199 (40.8)	289 (59.2)	
Age at enrolment (yrs)	51.52 (15.84)	51.26 (16.02)	51.76 (15.67)	0.28
SBP (mmHg)	124.00 (17.85)	122.45 (18.36)	125.45 (17.25)	<0.001
DBP (mmHg)	76.75 (9.75)	75.88 (9.68)	77.55 (9.75)	<0.001
Weight (kg)	73.07 (14.95)	66.15 (13.11)	79.55 (13.62)	<0.001
BMI (kg/m^2^)	27.53 (5.11)	27.08 (5.75)	27.95 (4.39)	<0.001
Kcalories	2173.05 (794.20)	1953.94 (716.36)	2378.46 (808.71)	<0.001
Wine (mL/day)	120.04 (175.23)	51.71 (94.48)	184.10 (206.51)	<0.001
TGs (mg/dL)	122.07 (87.59)	105.47 (70.17)	137.63 (98.75)	<0.001
TC (mg/dL)	197.29 (39.20)	198.21 (38.48)	196.44 (39.85)	0.12
HDL (mg/dL)	51.63 (13.63)	56.65 (13.95)	46.93 (11.49)	<0.001
LDL (mg/dL)	121.53 (33.66)	120.47 (33.48)	122.53 (33.80)	0.034
Glucose (mg/dL)	105.88 (25.26)	102.72 (24.94)	108.83 (25.21)	<0.001
GGT (U/L)	15.13 (15.06)	12.10 (13.42)	17.98 (15.93)	<0.001
ALT (U/L)	16.70 (13.40)	14.08 (11.44)	19.16 (14.58)	<0.001
AST (U/L)	12.29 (8.06)	11.43 (7.65)	13.10 (8.35)	<0.001
Smoking				
Never/former	4056 (83.3)	2079 (51.3)	1977 (48.7)	<0.001
Current	813 (16.7)	277 (34.1)	536 (65.9)	
Age at death (yrs)	69.05 (57.40–79.98)	69.33 (57.37–80.42)	68.93 (57.41–79.49)	0.82
Observation time (yrs)	18.80 (18.05–19.04)	18.80 (18.10–19.04)	18.81 (18.03–19.04)	0.067
Status				
Alive and/or censored	3841 (78.9)	1929 (50.2)	1912 (49.8)	<0.001
Dead	1028 (21.1)	427 (41.5)	601 (58.5)	
Cause of death				
GC	108 (10.5)	33 (30.6)	75 (69.4)	0.039
OCr	180 (17.5)	73 (40.6)	107 (59.4)	
DOC	740 (72.0)	321 (43.4)	419 (56.6)	
CCI	3.00 (1.00–4.00)	2.00 (1.00–4.00)	3.00 (1.00–5.00)	<0.001
Hypertension				
No	3664 (75.3)	1784 (48.7)	1880 (51.3)	0.46
Yes	1205 (24.7)	572 (47.5)	633 (52.5)	
Dyslipidemia				
No	4062 (83.4)	2024 (49.8)	2038 (50.2)	<0.001
Yes	807 (16.6)	332 (41.1)	475 (58.9)	
Diabetes				
No	4543 (93.3)	2229 (49.1)	2314 (50.9)	<0.001
Yes	326 (6.7)	127 (39.0)	199 (61.0)	
rMED				
rMED score				
Low	1286 (26.4)	492 (38.3)	794 (61.7)	<0.001
Medium	2561 (52.6)	1214 (47.4)	1347 (52.6)	
High	1022 (21.0)	650 (63.6)	372 (36.4)	
Job				
Managers and professionals	174 (6.2)	37 (21.3)	137 (78.7)	<0.001
Craft, agricultural, and sales workers	859 (30.4)	268 (31.2)	591 (68.8)	
Elementary occupations	738 (26.1)	300 (40.7)	438 (59.3)	
Homemaker	334 (11.8)	332 (99.4)	2 (0.6)	
Pensioners	680 (24.1)	251 (36.9)	429 (63.1)	
Unemployed	42 (1.5)	20 (47.6)	22 (52.4)	
Education				
Illiterate	167 (3.5)	90 (53.9)	77 (46.1)	<0.001
Primary school	1328 (27.5)	721 (54.3)	607 (45.7)	
Secondary school	1475 (30.5)	673 (45.6)	802 (54.4)	
High school	1374 (28.4)	645 (46.9)	729 (53.1)	
Graduate	487 (10.1)	207 (42.5)	280 (57.5)	
Marital status				
Single	755 (15.6)	378 (50.1)	377 (49.9)	<0.001
Married/cohabiting	3649 (75.5)	1640 (44.9)	2009 (55.1)	
Separated/divorced	117 (2.4)	70 (59.8)	47 (40.2)	
Widower	310 (6.4)	248 (80.0)	62 (20.0)	

**Abbreviations**: DBP: diastolic blood pressure; SBP: systolic blood pressure; BMI: body mass index; TGs: triglycerides; TC: total cholesterol; HDL: high-density lipoprotein cholesterol; LDL: low-density lipoprotein cholesterol; GGT: gamma glutamyl transpeptidase; ALT: alanine aminotransferase; AST: aspartate transaminase; GC: gastrointestinal cancer; OCr: other cancers; DOC: other causes death. CCI: Charlson Comorbidity Index. rMED: relative Mediterranean Scoring System. ^1^ as mean and standard deviation or median and interquartile range for continuous variables, and as frequency and percentage (%) for categorical. ^2^ Percentages calculated per column. ^3^ Percentages calculated per row. ^4^ Wilcoxon rank-sum test and Pearson’s chi-square tests were used to test differences between means and proportions, respectively.

**Table 2:** Mean weekly meat consumption (g/week) distributed by cause of death.

N (%)	Total Deaths	GC	OCr	DOC	*p*-Value ^1^
	1028	108 (10.5)	180 (17.5)	740 (72.0)	
	Mean (SE)	Mean (SE)	Mean (SE)	Mean (SE)	
rMED	8.74 (0.10)	8.59 (0.34)	8.44 (0.27)	8.83 (0.11)	
Total Meat (g/week)	371.13 (8.46)	411.74 (23.92)	407.90 (22.19)	356.56 (9.79)	0.020
Red Meat (g/week)	220.12 (5.95)	230.22 (15.86)	263.34 (18.49)	208.25 (6.48)	0.002
Lamb (g/week)	52.23 (3.27)	56.53 (10.42)	61.71 (123.87)	49.33 (3.64)	0.33
Horse (g/week)	22.73 (1.79)	22.44 (5.79)	27.36 (67.15)	21.69 (1.90)	0.50
Pig (g/week)	43.88 (1.76)	54.50 (5.40)	51.98 (70.19)	40.44 (1.92)	0.006
Calf (g/week)	106.65 (3.93)	102.58 (9.71)	129.43 (164.61)	101.72 (4.33)	0.028
White Meat (g/week)	151.01 (4.86)	181.53 (15.72)	144.56 (9.47)	148.31 (5.90)	0.10
Rabbit (g/week)	42.18 (2.15)	44.88 (5.90)	34.66 (3.65)	43.62 (2.71)	0.27
Poultry (g/week)	108.84 (3.82)	136.65 (12.72)	109.90 (8.10)	104.69 (4.55)	0.044

^1^ The Kruskal–Wallis test was used to determine the differences in means between specific causes of death and meat consumption. **Abbreviations:** SE: standard error; GC: gastrointestinal cancer; OCr: other cancers; DOC: other causes of death; rMED: relative Mediterranean Scoring System.

**Table 3:** Mortality hazard ratios (HRs) and subdistribution hazard ratios (SHRs) calculated according to the categories of meat, red meat, and poultry consumption (g/week).

	All Causes	GC	OCr	DOC
	HR (95% CI)	SHR (95% CI)	SHR (95% CI)	SHR (95% CI)
Model a				
Meat consumption (g/week)				
<200	1.00	1.00	1.00	1.00
200–300	0.81 * (0.67; 0.98)	0.46 * (0.23, 0.93)	0.82 (0.51; 1.32)	0.89 (0.72; 1.10)
301–400	1.09 (0.90; 1.32)	1.14 (0.64; 2.03)	1.19 (0.76; 1.86)	0.95 (0.76; 1.19)
>400	1.18 (0.99; 1.39)	1.23 (0.73; 2.06)	1.14 (0.76; 1.71)	1.00 (0.82; 1.22)
Model b				
Red meat consumption (g/week)				
<150	1.00	1.00	1.00	1.00
150–250	0.80 * (0.65; 0.98)	0.95 (0.48; 1.86)	0.71 (0.43; 1.18)	0.86 (0.68; 1.08)
251–350	0.97 (0.79; 1.19)	1.23 (0.63; 2.39)	0.79 (0.49; 1.29)	0.99 (0.78; 1.25)
>350	0.97 (0.79; 1.19)	1.05 (0.53; 2.09)	1.02 (0.65; 1.61)	0.88 (0.69; 1.12)
Model c				
Poultry consumption (g/week)				
<100	1.00	1.00	1.00	1.00
100–200	1.09 (0.94; 1.26)	1.65 * (1.06; 2.59)	0.90 (0.64; 1.27)	0.99 (0.83; 1.17)
201–300	1.13 (0.88; 1.45)	2.11 * (1.09; 4.09)	0.94 (0.54; 1.65)	0.97 (0.70; 1.34)
>300	1.27 * (1.00; 1.61)	2.27 * (1.23; 4.17)	0.93 (0.53; 1.61)	1.07 (0.80; 1.43)

* *p*-value < 0.05. Model a was adjusted for sex (women vs. men), gamma glutamyl transpeptidase, glucose, total cholesterol, smoke, hypertension, diabetes, dyslipidemia, wine, and relative Mediterranean Scoring System. Model b was adjusted for sex (women vs. men), gamma glutamyl transpeptidase, glucose, total cholesterol, smoke, hypertension, diabetes, dyslipidemia, wine, relative Mediterranean Scoring System, and white meat consumption. Model c was adjusted for sex (women vs. men), gamma glutamyl transpeptidase, glucose, total cholesterol, smoking, hypertension, diabetes, dyslipidemia, wine, relative Mediterranean Scoring System, and red meat consumption. **Abbreviations:** HR: hazard ratio; SHR: subdistribution hazard ratio; GC: gastrointestinal cancer; OCr: Other cancer deaths; DOC: deaths from other causes.

**Table 4:** Mortality hazard ratios (HRs) and subdistribution hazard ratios (SHR) calculated according to the categories of meat, red meat and poultry consumption (g/week) and sex.

	All Causes	GC	OCr	DOC
	HR (95% CI)	SHR (95% CI)	SHR (95% CI)	SHR (95% CI)
Model *d*				
Meat consumption (g/week)				
Men				
<200	1.00	1.00	1.00	1.00
200–300	0.73 * (0.56; 0.95)	0.32 * (0.13; 0.80)	1.02 (0.52; 2.00)	0.80 (0.61; 1.07)
301–400	1.01 (0.78; 1.29)	0.81 (0.40; 1.67)	1.58 (0.83; 3.01)	0.81 (0.61; 1.08)
>400	1.10 (0.88; 1.38)	1.00 (0.54; 1.84)	1.32 (0.73; 2.37)	0.89 (0.68; 1.15)
Women				
<200	1.00	1.00	1.00	1.00
200–300	0.92 (0.69; 1.23)	0.80 (0.26; 2.49)	0.69 (0.34; 1.40)	1.00 (0.72; 1.38)
301–400	1.24 (0.92; 1.67)	1.90 (0.74; 4.87)	0.85 (0.42; 1.71)	1.16 (0.81; 1.68)
>400	1.27 (0.97; 1.65)	1.66 (0.64; 4.27)	1.05 (0.58; 1.89)	1.17 (0.86; 1.57)
Model *e*				
Red meat consumption (g/week)				
Men				
<150	1.00	1.00	1.00	1.00
150–250	0.80 (0.60; 1.07)	1.03 (0.44; 2.39)	0.69 (0.31; 1.52)	0.84 (0.61; 1.15)
251–350	0.98 (0.74; 1.29)	1.05 (0.45; 2.44)	1.12 (0.55; 2.28)	0.92 (0.67; 1.26)
>350	0.92 (0.70; 1.22)	0.90 (0.38; 2.11)	1.26 (0.64; 2.48)	0.78 (0.57; 1.08)
Women				
<150	1.00	1.00	1.00	1.00
150–250	0.78 (0.59; 1.05)	0.64 (0.19; 2.12)	0.78 (0.41; 1.49)	0.86 (0.61; 1.21)
251–350	0.96 (0.71; 1.31)	1.75 (0.60; 5.11)	0.49 (0.23; 1.07)	1.02 (0.71; 1.48)
>350	1.06 (0.77; 1.44)	1.25 (0.41; 3.79)	0.87 (0.44; 1.69)	1.04 (0.71; 1.51)
Model *f*				
Poultry consumption (g/week)				
Men				
<100	1.00	1.00	1.00	1.00
100–200	1.06 (0.88; 1.28)	1.65 (0.95; 2.87)	1.02 (0.65; 1.58)	0.89 (0.72; 1.12)
201–300	1.01 (0.72; 1.41)	2.12 (0.96; 4.70)	0.86 (0.41; 1.78)	0.80 (0.51; 1.25)
>300	1.25 (0.93; 1.67)	2.61 * (1.31; 5.19)	0.70 (0.33; 1.51)	1.01 (0.69; 1.47)
Women				
<100	1.00	1.00	1.00	1.00
100–200	1.13 (0.89; 1.43)	1.37 (0.63; 2.99)	0.75 (0.42; 1.32)	1.16 (0.88; 1.52)
201–300	1.33 (0.90; 1.97)	1.72 (0.51; 5.82)	1.07 (0.43; 2.67)	1.20 (0.74; 1.94)
>300	1.34 (0.90; 2.00)	1.49 (0.35; 5.26)	1.40 (0.61; 3.18)	1.17 (0.72; 1.90)

* *p*-value < 0.05. Model d was adjusted for smoking, total cholesterol, GGT, glucose, marital status, hypertension, diabetes, dyslipidemia, wine, and relative Mediterranean Scoring System. Model e was adjusted for smoking, total cholesterol, gamma glutamyl transpeptidase, glucose, marital status, wine, relative Mediterranean Scoring System, and white meat consumption (g/week). Model f was adjusted for smoking, total cholesterol, GGT, glucose, marital status, wine, relative Mediterranean Scoring System and red meat consumption (g/week). **Abbreviations:** HR: hazard ratio; SHR: subdistribution hazard ratio; GC: gastrointestinal cancer; OCr: other cancer deaths; DOC: deaths from other causes.

## Data Availability

The original data presented in the study are openly available from FigShare at https://doi.org/10.6084/m9.figshare.28694684.

## Supplementary Materials

The following supporting information can be downloaded at: https://www.mdpi.com/article/10.3390/nu17081370/s1, Figure S1. Test of proportional hazards assumption for meat consumption categories (g/week). Figure S2. Test of proportional hazards assumption for red meat consumption categories (g/week). Figure S3. Test of proportional hazards assumption for red poultry consumption categories (g/week). Figure S4. LASSO for prediction and model selection. Table S1. Mean weekly meat consumption (g/week). Distributed by causes of death and gender.

## Abbreviations

The following abbreviations are used in this manuscript:

CIF	Cumulative Incidence Function
CRC	Colorectal cancer
DOC	Other causes of death
GC	Gastrointestinal cancer
HR	Hazard ratio
MICOL	Multicentric Italian Cholelithiasis
NUTRIHEP	Nutrition and Hepatology
OCr	Other cancers
SHR	Subdistribution hazard ratio
