# Supplementary material for: Does Poultry Consumption Increase the Risk of Mortality for Gastrointestinal Cancers? A Preliminary Competing Risk Analysis
Source: Nutrients. 2025 Apr 17;17(8):1370. doi: 10.3390/nu17081370 (PMC12030727; doi:10.3390/nu17081370)
Supplement: Supplementary file 1 [file nutrients-17-01370-s001.zip › nutrients-3591477-supplementary.pdf]

Does Poultry Consumption Increase the Risk of Mortality for Gastrointestinal Cancers? A Preliminary Competing Risk Analysis

Supplementary section

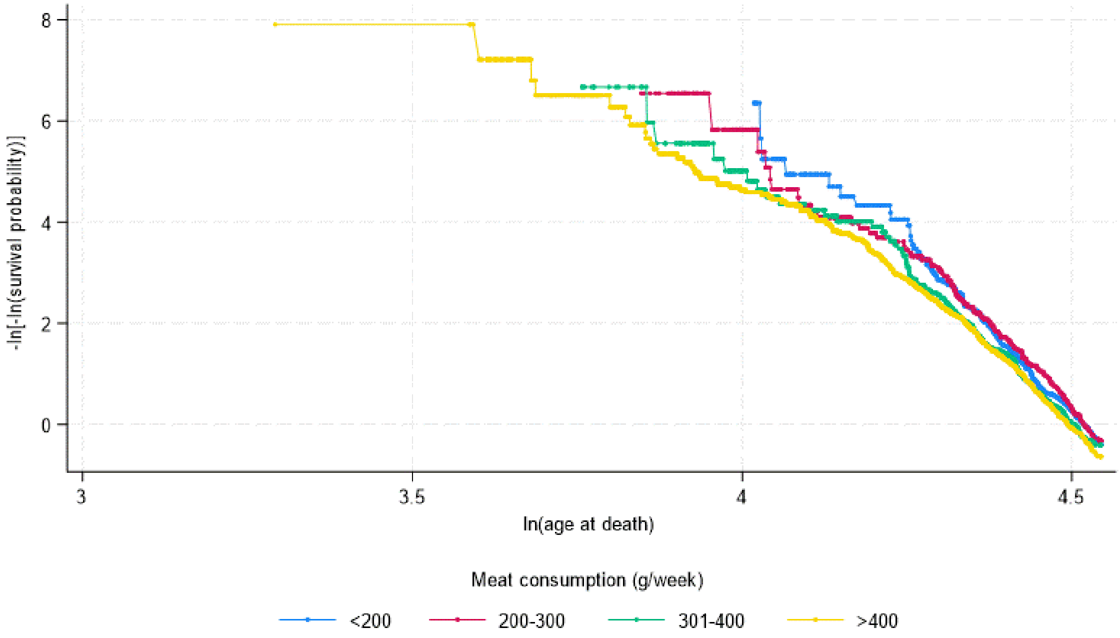

Test of proportional-hazards assumption

Time function: Rank of analysis time

	rho	chi2	df	Prob>chi2
0b.meatcate	.	.	1	.
1.meatcate	0.00648	0.04	1	0.8414
2.meatcate	-0.03653	1.27	1	0.2597
3.meatcate	-0.03050	0.88	1	0.3484
Global test		2.50	3	0.4749

Figure S1. Test of proportional-hazards assumption for meat consumption categories (g/week).

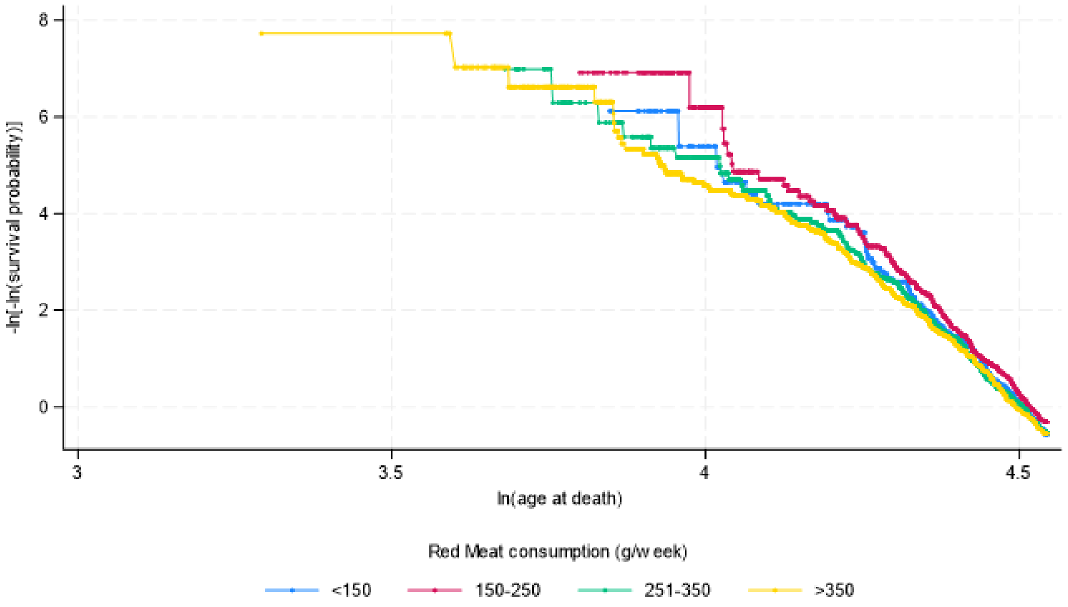

Test of proportional-hazards assumption

Time function: Rank of analysis time

	rho	chi2	df	Prob>chi2
0b.red_mea~t	.	.	1	.
1.red_meat~t	-0.00411	0.02	1	0.8988
2.red_meat~t	-0.03085	0.91	1	0.3408
3.red_meat~t	-0.04309	1.76	1	0.1852
Global test		2.88	3	0.4106

Figure S2. Test of proportional-hazards assumption for red meat consumption categories (g/week).

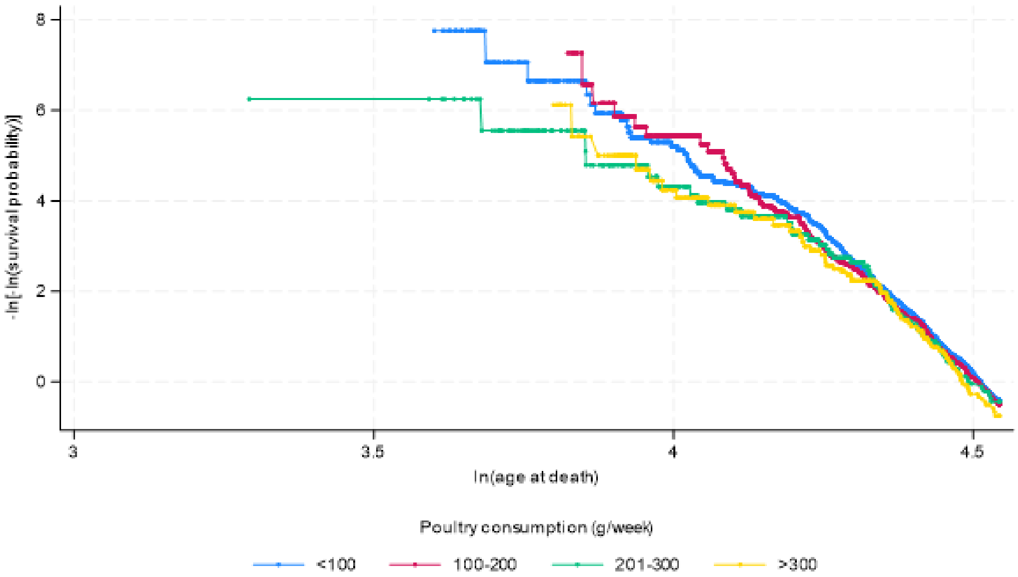

Test of proportional-hazards assumption

Time function: Rank of analysis time

	rho	chi2	df	Prob>chi2
0b.poultry~t	.	.	1	.
1.poultry_~t	-0.01387	0.18	1	0.6683
2.poultry_~t	-0.02107	0.42	1	0.5153
3.poultry_~t	0.00862	0.07	1	0.7900
Global test		0.70	3	0.8743

Figure S3. Test of proportional-hazards assumption for red poultry consumption categories (g/week).

Figure S4. Lasso for prediction and model selection.

Model a

	minBIC	adaptive
Smoke	x	x
gender	x	x
GGT	x	x
Cholesterol	x	x
Glucose	x	x
rMED_Score	x	x
meatcate	x	x
Marital_Status	x	x
Wine	x	x

Model b

	minBIC	adaptive
Smoke	x	x
gender	x	x
GGT	x	x
Cholesterol	x	x
Glucose	x	x
rMED_Score	x	x
white_meat	x	x
Marital_Status	x	x
Wine	x	x
red_meat_cat	x	x

Model c

	minBIC	adaptive
Smoke	x	x
gender	x	x
GGT	x	x
Cholesterol	x	x
Glucose	x	x
rMED_Score	x	x
poultry_cat	x	x
Marital_Status	x	x
red_week	x	x
Wine	x	x

Does Poultry Consumption Increase the Risk of Mortality for Gastrointestinal Cancers? A Preliminary Competing Risk Analysis

Table S1. Mean weekly meat consumption (g/week). Distributed by causes of death and gender.

	Female				*Male*			
	Total Death	CG	OCr	DOC	Total Death	CG	OCr	DOC
	Mean (SE)	Mean (SE)	Mean (SE)	Mean (SE)	Mean (SE)	Mean (SE)	Mean (SE)	Mean (SE)
rMED	8,86 (0,14)	8.96 (0.55)	8.72 (0.38)	8.88 (0.16)	8.62 (0.14)	8.52 (0.41)	8.07 (0.38)	8.78 (0.16)
Total Meat (g/week)	327.0 (12.6)	360.8 (38.96)	356.4 (35.31)	316.8 (14.24)	402.5 (11.17)	429.9 (28.4)	448.1 (28.53)	385.9 (13.28)
Red Meat (g/week)	186.6 (8.64)	198.3 (21.34)	219.5 (29.55)	178.0 (9.04)	243.9 (7.98)	240.4 (19.97)	293.9 (23.34)	231.7 (9.02)
Lamb (g/week)	43.1 (3.95)	36.03 (13.25)	53.97 (12.58)	41.38 (4.20)	58.70 (4.82)	62.83 (13.23)	67.32 (12.96)	55.76 (5.59)
Horse (g/week)	15.6 (1.93)	26.01 (9.96)	11.20 (3.48)	15.36 (2.18)	27.74 (2.71)	21.05 (6.81)	36.73 (9.00)	26.58 (2.90)
Pig (g/week)	36.8 (2.34)	44.76 (8.25)	35.52 5.39)	36.24 (2.74)	48.93 (2.49))	57.57 (6.59)	62.66 (7.84)	43.87 (2.68)
Calf (g/week)	94.9 (7.05)	96.21 (12.61)	122.2 (26.15)	88.60 (7.13)	115.0 (4.46)	104.8 (12.32)	135.8 (10.54)	111.5 (5.35)
White Meat (g/week)	140.4 (7.50)	162.6 (27.5)	137.0 (14.67)	138.9 (8.97)	158.6 (6.37)	189.5 (18.4)	154.2 (13.23)	154.1 (7.81)
Rabbit (g/week)	34.43 (2.44)	50.53 (13.99)	26.99 (4.51)	34.47 (2.71)	47.68 (3.22)	42.41 (5.51)	41.38 (5.37)	50.23 (4.29)
Poultry (g/week)	105.9 (6.34)	112.1 (17.95)	110.0 (13.87)	104.4 (7.62)	110.9 (4.73)	147.1 (15.84)	112.8 (10.77)	103.9 (4.73)

SE: Standard Error; GC: Gastrointestinal Cancer; OCr: Other Cancers; DOC: Other Causes Death; rMED: relative Mediterranean Scoring System.
